# Increase in Brain-Derived Neurotrophic Factor After Cycling Exercise Resisting Electrically Stimulated Antagonist Muscle Contractions in Overweight Japanese People: A Randomized, Controlled, Single-Blind, Crossover Trial

**DOI:** 10.7759/cureus.80694

**Published:** 2025-03-17

**Authors:** Takahiro Sugimoto, Ryuki Hashida, Sohei Iwanaga, Eriko Baba, Masayuki Omoto, Dan Nakano, Sachiyo Yoshio, Takumi Kawaguchi, Hiroo Matsuse

**Affiliations:** 1 Division of Rehabilitation, Kurume University Hospital, Kurume, JPN; 2 Division of Gastroenterology, Kurume University Hospital, Kurume, JPN; 3 Department of Human Immunology and Translational Research, National Center for Global Health and Medicine, Tokyo, JPN; 4 Division of Gastroenterology, Kurume University School of Medicine, Kurume, JPN

**Keywords:** aerobic exercises, cycle ergometer, myokine, neuromuscular electrical stimulation (nmes), obesity

## Abstract

Background: A hybrid training system (HTS) combining antagonist muscle electrical stimulation and voluntary muscle contraction has been developed using electrically stimulated eccentric antagonist muscle contractions. The exercise method that combines a conventional cycle ergometer with HTS (HERG) adds additional exercise intensity to the conventional cycle ergometer through electrical stimulation. Exercise-induced brain-derived neurotrophic factor (BDNF) production appears to have neuroprotective effects and contributes to improved metabolic regulation. Changes in BDNF after exercise are related to exercise intensity. Therefore, combining a cycle ergometer with electrical stimulation may be an effective approach.

Purpose: The purpose of this study was to evaluate the effect of the HERG on BDNF secretion.

Participants and methods: Fourteen healthy adults participated in the study. The participants performed two types of exercise at the anaerobic threshold: HERG and a cycling ergometer alone (CERG). A comparative study using a 2×2 crossover method was conducted to examine the differences in BDNF and lactate levels after HERG and CERG. A linear mixed model was used to compare changes in BDNF between HERG and CERG.

Results: Both HERG and CERG significantly increased BDNF and lactate levels after exercise. In overweight individuals with a BMI of 25 or higher, the change in BDNF levels after HERG was significantly greater than after CERG [ΔBDNF: 5500.96±7965.83 ng/ml, 1921.29±5308.22 ng/ml, respectively; p=0.0339]. There was no significant difference in the change in lactate levels after exercise between HERG and CERG (p=0.8632).

Conclusion: In overweight individuals, HERG increased post-exercise serum BDNF levels more than ergometer exercise alone, despite the exercise intensity remaining the same at the anaerobic threshold. The exercise method that combines the HERG may be a useful form of exercise for overweight individuals.

## Introduction

Aerobic exercises, such as using an ergometer, generally have high energy expenditure during the exercise and are effective in reducing body fat, increasing maximal oxygen uptake, improving exercise tolerance, and enhancing metabolic function. Furthermore, the ergometer is widely utilized as an exercise therapy not only for healthy individuals but also for those with relatively high exercise risks and low exercise tolerance, such as patients with musculoskeletal disorders, cardiovascular diseases, metabolic disorders, and frail elderly individuals [1-4]. This is because it allows for easy adjustment of exercise intensity and places less mechanical load on the lower joints compared with other types of aerobic exercises [5]. Therefore, ergometer exercise is particularly beneficial for individuals who require exercise, such as those with chronic conditions or overweight (obesity) at risk of joint disorders. These exercise benefits are more effective with higher exercise intensity [6]. However, when exercise tolerance is low, engaging in relatively high-intensity exercise can be challenging. Therefore, there is a need for exercise methods that are both feasible and more effective for patients with reduced exercise tolerance.

The mechanisms underlying the effects of exercise include the influence of myokines secreted by skeletal muscles during physical activity. One of these myokines is brain-derived neurotrophic factor (BDNF) [7]. In recent years, BDNF has been shown to have a relationship with metabolic function [7,8] and nutritional status [9]. BDNF secreted from skeletal muscles during exercise is considered an important factor in improving mitochondrial function [10]. In individuals with metabolic syndrome, the increase in BDNF levels after exercise has been shown to be associated with exercise benefits, including improvements in muscle strength [11], insulin resistance [12], and depressive symptoms [13]. BDNF has been shown to acutely increase immediately after exercise in the short term and to sustain elevated levels with regular exercise in the long term, potentially contributing to improvements in glucose and lipid metabolism induced by exercise [14]. Therefore, exercise that effectively stimulates sufficient BDNF secretion is preferable for individuals with metabolic syndrome or those who are overweight and at high risk of developing it. However, the magnitude of change in BDNF levels after aerobic exercise depends on the intensity of the exercise [15]. The increase in BDNF after exercise is observed in both obese and non-obese individuals. However, since the total relative energy expenditure at the same aerobic exercise intensity is significantly lower in obese individuals compared to non-obese individuals, the post-exercise BDNF response at a similar exercise intensity may be insufficient for improving insulin resistance or carbohydrate and fat utilization in obese individuals [16]. Therefore, obese individuals with low exercise tolerance may find it challenging to engage in exercise at a sufficient intensity to induce effective changes in BDNF levels after exercise. Thus, exercises that can increase BDNF levels even at relatively low intensity would be beneficial.

It has been reported that combining neuromuscular electrical stimulation (NMES) with voluntary contractions is more effective than using either NMES or voluntary contractions alone [17]. The hybrid training system (HTS) is an exercise that combines NMES with voluntary contractions simultaneously during exercises [18]. Using an ergometer for aerobic exercise has been tested and shown to work well with NMES [19]. We reported that oxygen uptake could be increased during ergometer exercise without increasing the workload by combining it with NMES [20]. Furthermore, it has been reported that HTS can improve insulin resistance and liver function in patients with non-alcoholic fatty liver disease [21]. Therefore, performing HTS simultaneously during exercise may be effective in stimulating BDNF secretion. It has also been reported that higher BMI is associated with BDNF secretion after exercise [21]. Consequently, it is hypothesized that a cycling ergometer combined with HTS (HERG) would be particularly effective as a stimulus for BDNF secretion in individuals with a higher BMI. In this study, we investigated the immediate effect of HERG on serum BDNF levels after exercise, taking into account the influence of BMI. By examining the immediate increase in BDNF after HERG in this study, we indirectly assess the potential long-term exercise effects of HERG.

## Materials and methods

Participants

Fifteen healthy young men (mean±standard deviation (SD): age 29.4±6.5 years; height 170.0±6.1 cm; body mass 68.2±8.9 kg) agreed to participate. All participants were given an explanation of the purpose and methods of the study, both verbally and in writing, and written consent to participate in this study was obtained. The inclusion criteria were men aged 20 years or older, with no current medical conditions requiring treatment and no limitations in daily activities.

The exclusion criteria included a history of musculoskeletal disorders, cardiovascular disease, respiratory disease, neuromuscular disorders, and neuropsychiatric disorder. All participants first underwent a medical examination and cardiopulmonary exercise tests (CPX) conducted by a physician and/or physical therapist, followed by the completion of two different types of exercise sessions in a randomized order, as described later. The study was conducted in adherence with the standards of the World Medical Association Declaration of Helsinki: Ethical Principles for Medical Research Involving Human Subjects (2013 version). The Ethics Committee of Kurume University (Health Care and Medical Ethics) approved the clinical design of this study protocol (approval ID: 19055). This study was registered on the UMIN Clinical Trials Registry (UMIN000055280). The link to this study data is https://dataverse.harvard.edu/dataset.xhtml?persistentId=doi:10.7910/DVN/BYVT3U.

Study design

**Figure 1:**
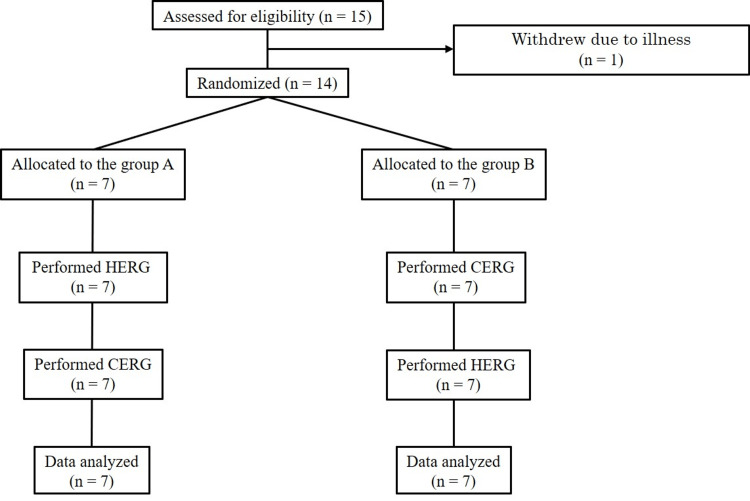
Diagram of participants’ flow throughout the study. HERG, conventional cycle ergometer with HTS; CERG, cycling ergometer alone.

This study is a comparative research design using a 2×2 crossover method. Each participant performed two types of exercise: HERG and cycling ergometer alone (CERG) (Figure 1). Group A performed HERG first, while Group B performed CERG first. Participants were randomly assigned to Group A or Group B by staff members not involved in the study using a 1:1 random number generator sequence generated prior to the initiation of the study (Randomization.com). To ensure allocation concealment, computer-generated randomization and the envelope method were used. The participants were not blinded as they were aware of the type of exercise they would perform, but assessors and analysts were blinded. The washout period between the two types of exercise interventions (CERG or HERG) was set at one week, and participants were assessed (blood test) immediately before and after each exercise intervention. This study was designed in accordance with the CONSORT guidelines (www.consort-statement.org.) for reporting randomized controlled trials (Appendix, Table 3).

Setting

The study was conducted in a rehabilitation room affiliated with the university hospital.

Ramp load test

To set the exercise load, CPX (Mobile aeromonitor AE-100i, Minato Medical Science Co., Ltd., Osaka, Japan) were performed to measure the anaerobic threshold (AT) and peak oxygen uptake (VO_2_peak). The ramp load test protocol was conducted based on our previous report [22]. The heart rate at the AT was set as the target heart rate for the exercise session.

CERG protocol

After a two-minute warm-up at 30W, participants performed ergometer exercise for 30 minutes at an exercise intensity corresponding to their AT. During the exercise, participants were instructed and monitored to maintain a cadence of 60-80 revolutions per minute and to maintain their target heart rate.

HERG protocol

**Figure 2:**
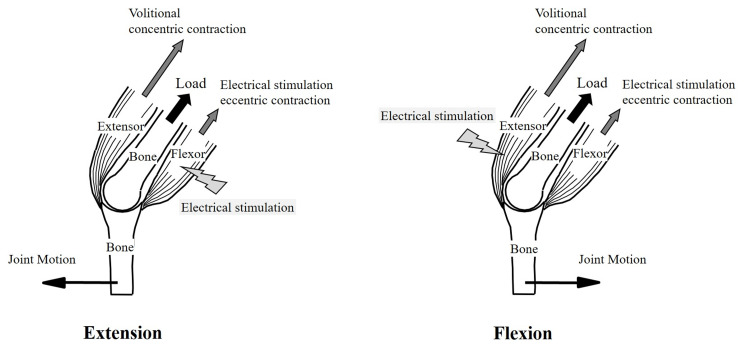
HTS. HTS creates resistance to the motion of voluntarily contracting agonist muscles through the force generated by its electrically stimulated antagonist muscles. In this process, electrical stimulation induces eccentric contractions in the antagonist muscles, while voluntary muscle contractions result in concentric contractions in the agonist muscles. HTS combines the effects of both electrical stimulation and voluntary movement. This figure was adapted from [19]. HTS: hybrid training system.

NMES was combined with ergometer exercise simultaneously [18]. During HTS with the ergometer, as in previous studies, electrical stimulation was set so that the hamstrings were stimulated during knee extension and the quadriceps during knee flexion (Figure 2) [19]. After a two-minute warm-up at 30W, participants performed the ergometer exercise for 30 minutes. They were instructed to maintain a cadence of 60-80 revolutions per minute. In a previous study, we reported that the oxygen uptake during HERG has a linear relationship with workload similar to a conventional ergometer and that exercise intensity could be adjusted using heart rate [20]. The oxygen uptake during HERG was significantly higher than during CERG at an average of 21.2%. Based on these data, the workload was calculated (target heart rate) using a linear regression equation and adjusted during HERG to reach the same oxygen uptake as that attained during CERG. Therefore, the ratio of oxygen uptake to VO_2_peak in both the HERG and CERG groups was the same, at 40%.

HTS protocol

The stimulation waveform used a carrier frequency of 5,000 Hz, with a frequency of 40 Hz (2.4 ms on, 22.6 ms off), a pulse width of 200 μs, and a biphasic rectangular waveform. The electrical stimulation intensity was set at 80% of the maximum tolerable voltage, a level that previous studies have shown to be effective for improving muscle strength and mass while ensuring safety [18]. The electrical stimulation device used was the HTS from Panasonic Corporation (Home Appliance Development Center, Corporate Engineering Division, Appliance Company, Panasonic Corporation, Kusatsu City, Shiga, Japan). An accelerometer (EWTS9PD, Home Appliance Development Center, Corporate Engineering Division, Appliance Company, Panasonic Corporation, Kusatsu City, Shiga, Japan) was placed on the front of each leg to serve as a joint motion sensor. The motion sensor measures the hip joint angular velocity during pedaling, and the quadriceps or hamstrings are electrically stimulated in sync with the movement of the knee joint during pedaling. A pair of 5x12 cm low-impedance gel-coated silver fiber electrodes (Nihon Medix Co., Matsudo City, Chiba, Japan) were applied to the motor points of the quadriceps and hamstrings on both legs.

Blood sampling

Participants were instructed to fast and abstain from alcohol and caffeine for 12 hours before the exercise session. They were also advised to refrain from any sports activities for one week prior to the study and during the study period. To avoid circadian fluctuations in metabolism and hormone levels, the exercise interventions were conducted between 7:00 and 8:00 AM. Blood samples were drawn from the superficial veins of the forearm at two times: before exercise and immediately after exercise. Blood lactate concentration values were measured by finger prick using the Lactate Pro 2 (LT-1730, Arkray, Co., Kyoto City, Kyoto, Japan). After blood collection, the blood was centrifuged at 3000 rpm for 10 minutes, and the serum was stored at -80°C. For the analysis of BDNF, a Human Free BDNF Quantikine ELISA Kit (R&D Systems, Minneapolis, MN, USA) was used to analyze human serum samples.

Statistical analysis

Changes before and after each exercise (HERG and CERG) were compared using the Wilcoxon signed-rank test. The changes after each exercise were compared using a linear mixed model based on a 2x2 crossover design. In this study, individuals with a BMI of 25 or higher (considered overweight by WHO standards) were defined as obese according to Japanese standards. Additionally, the prevalence of metabolic syndrome significantly increases in overweight men with a BMI of 25 or higher [23]. The analyzed data were presented as mean±standard deviation. Since this was a preliminary study, a sample size was not set. Therefore, as an exploratory study, the effect size (Cohen’s d) and statistical power regarding BDNF, the primary outcome, were calculated based on the results of this study. All statistical analyses were performed by JMP Version 16.0 statistical software (SAS Institute Inc., Cary, NC, USA), and values of p<0.05 were considered to be statistically significant.

## Results

Fifteen participants were initially enrolled in the study, but one withdrew due to illness just after the baseline assessment. Fourteen participants (age 29.1±6.6 years; height 170.0±6.2 cm; body mass 68.6±9.1 kg, BMI 23.6±2.7) completed the entire study. The characteristics of the participants are shown in Table 1. Of 14 individuals, six had a BMI of 25 or higher, classifying them as obese. No adverse events due to the intervention were observed.

**Table 1:** Characteristics of the participants. BMI, body mass index; N/A, not applicable; SD, standard deviation; VO_2_, oxygen uptake.

	Mean±SD
Number	N/A
Age (years）	29±6.6
Body weight (kg)	68.6±9.1
Height (cm)	170.0±6.2
BMI (kg/m^2^)	23.6±2.7
VO_2_ peak (ml/kg/min)	31.3±4.2

**Figure 3:**
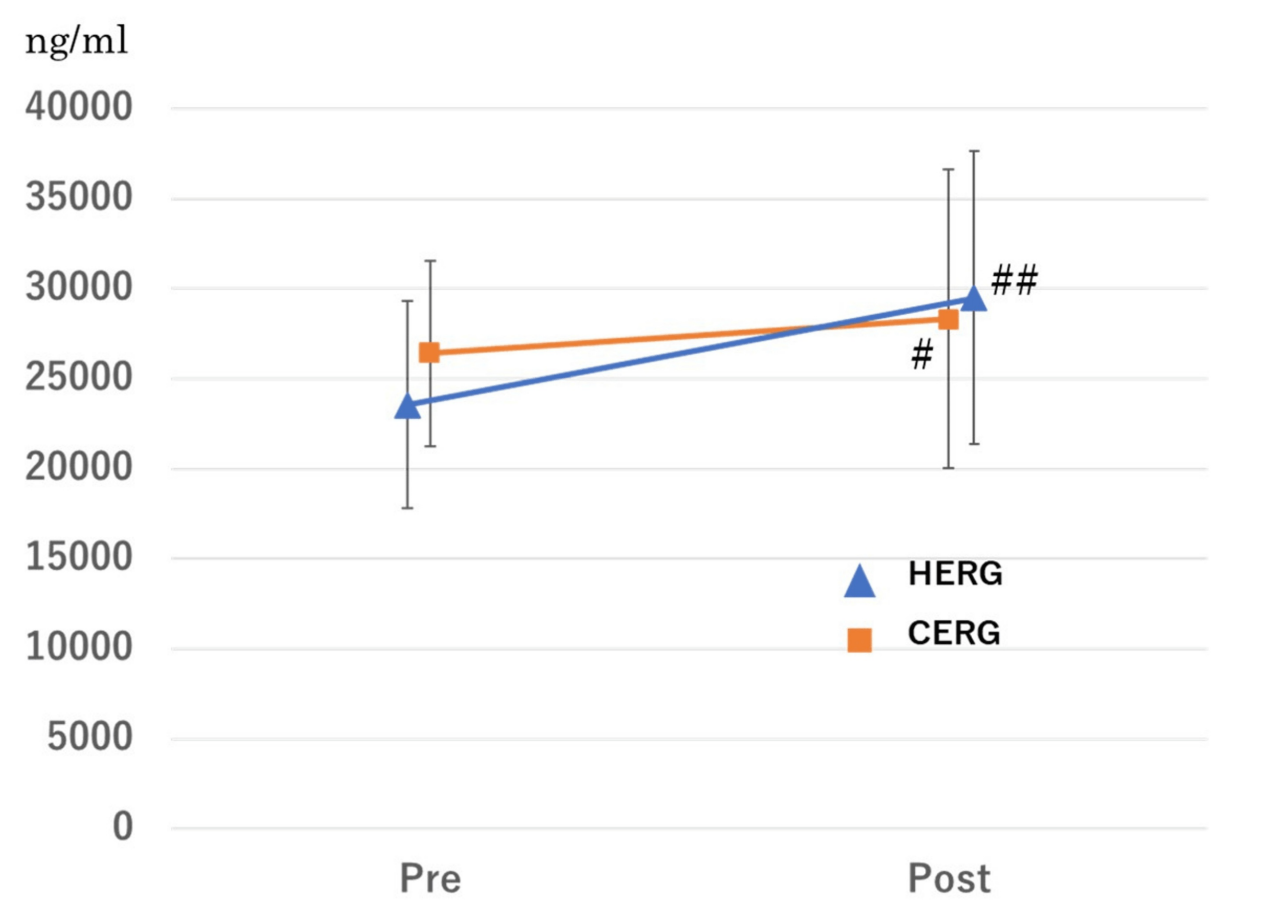
Changes of BDNF after HERG and CERG. BDNF, brain-derived neurotrophic factor; HERG, conventional cycle ergometer with HTS; CERG, cycling ergometer alone; HTS, hybrid training system. p-value is analyzed using a Wilcoxon signed-rank test. ^#^p<0.05, ^##^p<0.01.

Serum BDNF levels significantly increased after CERG from 26419.6±5161.9 ng/ml to 28340.8±8273.9 ng/ml (p=0.0259) (Figure 3). Similarly, serum BDNF levels significantly increased after HERG from 24294.9±5313.7 ng/ml to 29795.9±7519.9 ng/ml (p=0.0017) (Figure 3). The effect sizes (Cohen's d) for CERG and HERG were 0.28 and 0.85, respectively. The statistical power for HERG was 0.84.

The results of a linear mixed model showed that the type of exercise and the presence of overweight were significant factors associated with the change in BDNF levels after exercise. In obese individuals with a BMI of 25 or higher, the change in BDNF levels after HERG was significantly greater than after CERG [ΔBDNF: 5501.0±7965.8 ng/ml, 1921.3±5308.2 ng/ml, respectively; p=0.0339] (Table 2). A significant increase in lactate levels was observed after CERG from 1.45±0.46 mmol/l to 2.16±1.31 mmol/l (p=0.0381). A significant increase in lactate levels was observed after HERG from 1.39±0.52 mmol/l to 2.22±1.17 mmol/l (p=0.0093). However, in overweight individuals (obesity) with a BMI of 25 or higher, there was no significant difference in the change in lactate levels after exercise between HERG and CERG (p=0.8632) (Table 2).

**Table 2:** Changes of BDNF and lactate. BDNF, brain-derived neurotrophic factor; HERG, conventional cycle ergometer with HTS; CERG, cycling ergometer alone; HTS, hybrid training system. p-value is analyzed using a linear mixed model based on a 2x2 crossover design. ^#^Overweight was defined as a BMI of 25 or higher, and adjustments were made based on the presence or absence of overweight.

	HERG	CERG	p-value^#^
BDNF (ng/ml)	Δ5501.0±7963.8	Δ1921.3±5308.2	p=0.0339
Lactate (mmol/l)	Δ0.85±1.2	Δ0.73±1.4	p=0.8632

## Discussion

We investigated the effects of a combined exercise method involving ergometer and NMES (HTS) on post-exercise serum BDNF levels. Regardless of the presence of NMES, serum BDNF levels significantly increased after ergometer exercise at the AT level. Additionally, in individuals with overweight (BMI ≥ 25), despite exercising at the same intensity, the ergometer combined with NMES (HERG) significantly increased post-exercise serum BDNF levels more than the conventional ergometer (CERG). For individuals with overweight, combining NMES with ergometer exercise might be a more effective and accessible exercise method compared to conventional ergometer exercise.

The magnitude of the change in BDNF after exercise is related to the exercise intensity [15]. In this study, it is believed that exercise intensity influenced the change in BDNF after exercise. The target heart rates during exercise for both the HERG and CERG groups were comparable, and there was no difference in the change in lactate levels after exercise, suggesting that the aerobic exercise intensity was equivalent. HERG may have imposed a higher exercise load than the conventional ergometer (CERG). HTS allows for the simultaneous performance of resistance exercise using electrical stimulation during aerobic exercise [18]. HTS during aerobic exercise not only provides the benefits of aerobic exercise but also promotes muscle strength and hypertrophy [19]. Changes in BDNF after exercise are observed not only following aerobic exercise but also after resistance exercise. However, resistance training aimed at muscle hypertrophy to the point of fatigue has been shown to be particularly effective [24]. Therefore, the localized resistance exercise load from HTS may have contributed to the greater increase in BDNF after HERG. Additionally, the increase in BDNF after exercise has been shown to be more effective with interval training than with continuous exercise [25]. The electrical stimulation during HERG is intermittent because the flexors and extensors are alternately electrically stimulated in response to voluntary flexion and extension movements, making it a form of interval training that utilizes electrical stimulation. These factors, such as the mechanical intensity and the interval training characteristics, may have contributed to the greater increase in BDNF after HERG. Thus, combining aerobic exercise with NMES simultaneously (HTS) is believed to effectively stimulate BDNF secretion by increasing the exercise load (mechanical load) even at the same exercise intensity (oxygen uptake). Additionally, although HERG provides the same overall aerobic exercise intensity as CERG, the pedal load is lower due to the HTS, making it easier for individuals with lower fitness levels, including those who are overweight, to pedal comfortably.

NMES has been shown to increase BDNF levels comparable to those observed after moderate-intensity aerobic exercise [26]. On the other hand, Kimura et al. reported that NMES (1.41 METs) significantly increased BDNF levels compared to quadriceps isometric resistance exercise [27]. These reports suggest that NMES appears to increase BDNF levels even at lower intensities compared to typical aerobic or resistance exercises. Furthermore, the impact of electrical eccentric muscle contractions during HERG should also be considered. Responses from satellite cells, which are involved in muscle hypertrophy, have been observed after eccentric exercise [28], and it has been shown that these responses are associated with changes in BDNF after eccentric exercise [29]. Additionally, eccentric muscle contractions are useful for improving body composition, physical function, and metabolic health in patients with type 2 diabetes and obesity due to their ability to produce high muscle tension with lower energy expenditure [30]. Therefore, the significance of electrical eccentric muscle contractions was likely greater for obese patients compared to non-obese individuals and effective in stimulating post-exercise BDNF secretion even though the exercise intensity is relatively low. However, the results of this study do not clarify the mechanism. Furthermore, fundamental research is needed to verify the mechanisms underlying the effects on metabolic function mediated by changes in BDNF. Additionally, long-term intervention studies are necessary to demonstrate the sustained effects of this exercise.

Limitations

The effect size (0.85) and statistical power (0.84) of the HERG intervention were favorable, and its effectiveness considering BMI was demonstrated statistically. However, there were only six participants with a BMI of 25 or higher, which was not a large number. Further studies, including a higher proportion of overweight (obesity) individuals with a BMI of 25 or higher, are needed. Since BDNF is influenced by cognitive function, physical function, and muscle mass, it is also important to investigate its effects in older adults. Additionally, BDNF has been reported to be associated with heart failure, dementia, Parkinson's disease, and depression. Therefore, it is necessary to consider not only BMI but also those factors. Additionally, in this study, obesity was defined as a BMI of 25 or higher for Japanese. However, the WHO defines obesity as a BMI of 30 or higher, so it is necessary to investigate cases with a higher BMI. Furthermore, since the subjects of this study are all male and young, future research is needed involving females and a broader age range. It is also necessary to adjust for habitual nutrition intake and physical activity levels, which are believed to influence BDNF levels after exercise. While this study was conducted on healthy individuals, it will be necessary to verify the effects of HERG on patients with various conditions in the future. Moreover, in this study, BDNF was measured only at one time point immediately after exercise, so the timing of peak values and the sustained increase in BDNF remain unknown. Future studies should repeatedly measure BDNF over a period of time after exercise. Of course, long-term intervention studies are also necessary to verify the long-term effects of HERG.

## Conclusions

In individuals who are overweight, the combination of ergometer exercise and HTS increased post-exercise serum BDNF levels more than ergometer exercise alone. Combining ergometer exercise with NMES may be a beneficial form of exercise for individuals who are overweight.

## Appendices

**Table 3:** CONSORT 2010 checklist of information to include when reporting a randomized trial. ^*^We strongly recommend reading this statement in conjunction with the CONSORT 2010 Explanation and Elaboration for important clarifications on all the items. If relevant, we also recommend reading CONSORT extensions for cluster randomized trials, non-inferiority and equivalence trials, non-pharmacological treatments, herbal interventions, and pragmatic trials. Additional extensions are forthcoming: for those and for up-to-date references relevant to this checklist, see www.consort-statement.org.

Section/topic	Item no.	Checklist item	Reported on page no.
Title and abstract			
	1a	Identification as a randomized trial in the title	P1
	1b	Structured summary of trial design, methods, results, and conclusions (for specific guidance, see CONSORT for abstracts)	P1
Introduction			
Background and objectives	2a	Scientific background and explanation of rationale	P4-1 to P6-18
	2b	Specific objectives or hypotheses	P6-10 to P6-18
Methods			
Trial design	3a	Description of trial design (such as parallel, factorial), including allocation ratio	P7-21 to P8-11
	3b	Important changes to methods after trial commencement (such as eligibility criteria), with reasons	N/A
Participants	4a	Eligibility criteria for participants	P6-20 to P7-8
	4b	Settings and locations where the data were collected	P8-13
Interventions	5	The interventions for each group with sufficient details to allow replication, including how and when they were actually administered	P8-22 to P11-9
Outcomes	6a	Completely defined pre-specified primary and secondary outcome measures, including how and when they were assessed	P10-20 to P11-9
	6b	Any changes to trial outcomes after the trial commenced, with reasons	N/A
Sample size	7a	How sample size was determined	P11-20,21
	7b	When applicable, explanation of any interim analyses and stopping guidelines	N/A
Randomization:			
Sequence generation	8a	Method used to generate the random allocation sequence	P8-2 to P8-11
	8b	Type of randomization; details of any restriction (such as blocking and block size)	P8-2 to P8-11
Allocation concealment mechanism	9	Mechanism used to implement the random allocation sequence (such as sequentially numbered containers), describing any steps taken to conceal the sequence until interventions were assigned	P8-2 to P8-11
Implementation	10	Who generated the random allocation sequence, who enrolled participants, and who assigned participants to interventions	P8-2 to P8-11
Blinding	11a	If done, who was blinded after assignment to interventions (for example, participants, care providers, those assessing outcomes) and how	N/A
	11b	If relevant, description of the similarity of interventions	N/A
Statistical methods	12a	Statistical methods used to compare groups for primary and secondary outcomes	P11-12 to P12-2
	12b	Methods for additional analyses, such as subgroup analyses and adjusted analyses	P11-12 to P12-2
Results			
Participant flow (a diagram is strongly recommended)	13a	For each group, the numbers of participants who were randomly assigned, received intended treatment, and were analyzed for the primary outcome	P12-5-10
	13b	For each group, losses and exclusions after randomization, together with reasons	P12-5-10
Recruitment	14a	Dates defining the periods of recruitment and follow-up	Figure 2
	14b	Why the trial ended or was stopped	N/A
Baseline data	15	A table showing baseline demographic and clinical characteristics for each group	Table1
Numbers analyzed	16	For each group, number of participants (denominator) included in each analysis and whether the analysis was by original assigned groups	P12-5-8, Figure3
Outcomes and estimation	17a	For each primary and secondary outcome, results for each group, and the estimated effect size and its precision (such as 95% confidence interval)	P13-14 to P13-5
	17b	For binary outcomes, presentation of both absolute and relative effect sizes is recommended	P13-14-16
Ancillary analyses	18	Results of any other analyses performed, including subgroup analyses and adjusted analyses, distinguishing pre-specified from exploratory	P12-23 to P13-5, Table2
Harms	19	All important harms or unintended effects in each group (for specific guidance, see CONSORT for harms)	P12-10
Discussion			
Limitations	20	Trial limitations, addressing sources of potential bias, imprecision, and, if relevant, multiplicity of analyses	P16-2 to P17-3
Generalizability	21	Generalizability (external validity, applicability) of the trial findings	P13-8–17
Interpretation	22	Interpretation consistent with results, balancing benefits and harms, and considering other relevant evidence	P13-18 to P15-23
Other information			
Registration	23	Registration number and name of trial registry	P7-17
Protocol	24	Where the full trial protocol can be accessed, if available	P7-17
Funding	25	Sources of funding and other support (such as supply of drugs), role of funders	P18-6,7
